# Draft Genome Sequence of *Leuconostoc citreum* CW28 Isolated from Pozol, a Pre-Hispanic Fermented Corn Beverage

**DOI:** 10.1128/genomeA.01283-17

**Published:** 2017-11-30

**Authors:** Clarita Olvera, Rosa I. Santamaría, Patricia Bustos, Cristina Vallejo, Juan J. Montor, Carmen Wacher, Agustín Lopez Munguia

**Affiliations:** a Departamento de Ingeniería Celular y Biocatálisis, Instituto de Biotecnología, Universidad Nacional Autónoma de México, Cuernavaca, Morelos, Mexico; b Centro de Ciencias Genómicas, Universidad Nacional Autónoma de México, Cuernavaca, Morelos, Mexico; c Facultad de Química, Universidad Nacional Autónoma de México, CDMX, Mexico

## Abstract

*Leuconostoc citreum* CW28 was isolated from pozol, a Mayan fermented corn beverage. This strain produces a cell-associated inulosucrase, the first described in bacteria. Its draft genome sequence, announced here, has an estimated size of 1.98 Mb and harbors 1,915 coding genes, 12 rRNAs, 68 tRNAs, 17 putative pseudogenes, and 1 putative phage.

## GENOME ANNOUNCEMENT

Pozol is a nonalcoholic lactic acid beverage of Mayan origin obtained from the natural fermentation of nixtamal (heat- and alkali-treated maize). After lime treatment, the corn kernels are dehulled, washed, and ground, and the resulting dough is shaped into balls that are wrapped in banana leaves, which are fermented at room temperature for 2 days (1). The predominant bacterial groups during all stages of pozol fermentation are lactic acid bacteria (LAB), including *Lactobacillus*, *Weissella*, and *Leuconostoc* spp. (2). It has been demonstrated that some of the LAB involved in pozol fermentation may have probiotic properties and produce oligosaccharides (OS) and polysaccharides with potential biotechnological applications.

*Leuconostoc citreum* CW28, a strain isolated from pozol, produces high-molecular-weight (HMW) inulin from sucrose through a cell-associated multidomain inulosucrase, IslA (EC 2.4.1.9). The HMW inulin may be directly used as soluble fiber or hydrolyzed to obtain fructooligosaccharides (FOS) and oligofructose (3, 4). Until now, 9 genomes of *L. citreum* have been reported, including the complete genome of strain KM20, isolated from kimchi (5); draft genomes of strains LBAE E16, LBAE C10, and LBAE C11, isolated from wheat sourdough (6); strains NRRL B-742 and B-1299 (7); strain DmW_111, isolated from wild *Drosophila* (8); and two strains, 1300_LCIT and 1301_LGAS, isolated from intensive care units (9).

*L. citreum* CW28 was grown in MRS medium at 30°C (4). The genomic DNA was prepared using the phenol-chloroform method and sequenced in an Illumina HiSeq 2000 sequencer at Macrogen (Seoul, South Korea). Paired-end reads of 101 bp were assembled using SPAdes 3.5.0 (10), SSPACE-Basic 2.0 (11), and Consed v.23 (12), with a coverage of 80×. The contigs obtained were aligned to the nearest reference genome (*Leuconostoc citreum* KM20) using the NUCmer program (13). Open reading frames (ORFs) were predicted with Glimmer 3.0 (14); manual annotations were carried out in Artemis 12.0 (15), with comparisons with the GenBank (16), Interpro (17), and ISfinder (http://www-is.biotoul.fr) databases. The draft genome of *L. citreum* CW28 contains nine contigs, with a total length of 1,982,921 bp and 38.7% G+C content. A total of 1,915 protein-coding sequences (CDSs), 12 rRNAs, 68 tRNAs, and 17 putative pseudogenes were identified. The PHAST server (18) and manual annotation revealed one putative intact prophage of 34.8 kb with 37 CDSs. An analysis of similarity among the *L. citreum* genomes, measured as average nucleotide identities (ANI), shows that the genome of the *L. citreum* CW28 strain is more similar to B-1299, B-742, and LBAE E16 (99.42, 99.41, and 99.30% similar, respectively) than to KM20, LBAE C11, and LBAE C10 (98.57, 98.47, and 98.33% similar, respectively). Extensive genome analysis of this and other *L. citreum* strains will facilitate the identification of new genes encoding enzymes involved in polymer and OS synthesis with potential biotechnological applications.

### Accession number(s).

This whole-genome shotgun project has been deposited at DDBJ/EMBL/GenBank under the accession no. MWJP00000000. The version described in this paper is the first version, MWJP01000000.
